# The ultra-photostable and electrically modulated Stimulated Emission in perylene-based dye doped liquid crystal

**DOI:** 10.1038/s41598-019-38484-z

**Published:** 2019-02-14

**Authors:** A. Adamow, L. Sznitko, E. Chrzumnicka, J. Stachera, A. Szukalski, T. Martynski, J. Mysliwiec

**Affiliations:** 1 The Advanced Materials Engineering and Modelling Group, Wroclaw University of Science and Technology, Wybrzeze Stanislawa Wyspianskiego 27, 50-370 Wroclaw, Poland; 2 Institute for Materials Research and Quantum Engineering, Faculty of Technical Physic, Poznan University of Technology, Piotrowo 3, 60-965 Poznan, Poland

## Abstract

One of the most important drawback of organic dyes is their low photo-stability which reduces possibility of their commercial utilization. In this article we employ the strategy of dye re-crystallization from oversaturated matrix in order to enhance material’s durability. One of the main advantages of perylene derivative is ability to form emissive *j*-aggregates, good miscibility and incorporation into liquid crystalline matrix. Investigation of perylene-based dye and LC matrix brought as the result very efficient light amplification modulation by applied external electric field. In our article we show that Stimulated Emission (STE) is possible to achieve from perylene-derivative based system, at typical fluence thresholds for laser dyes: 3.9 mJ/cm^2^. Moreover, presented system proves ultra-high photostability, showing lack of STE reduction even after 12 000 excitation laser pulses. Furthermore, we proved the possibility of light emission intensity control using external electric field.

## Introduction

Organic dyes possess many interesting features that make them attractive for optical purposes. They show optically induced birefringence^1^, multiphoton phenomena^2^, they can be used as the laser dyes^3^ as well as nonlinear optical features activators for the inert matrices^4^. Desired optical features can be easily achieved using many well-known processing, combining the active matter associated with valuable features and - in general - inert/passive matrix^5^. The most important drawback that limits their utilization in commercial applications, especially when exploited with intense laser light, is their low photo-stability^6^. Certain efforts have been undertaken in order to avoid or limit photo-degradation process by branching dyes into the polymeric structures. Among them was, e.g. limiting the dye diffusion from excited area^7^ by incorporating to organic matrix also inorganic materials like Polyhedral Oligomeric Silsesquioxane (POSS) derivatives. It can form cage structures which increase heat dissipation through the increased rigidity of matrix and also prevent dye from photo-oxidation processes by creating steric collapse^8^. Another way to improve dye photostability can be achieved by simple encapsulation to reduce molecular oxygen appearance into the material^9^. Nevertheless, another approaches and trials to achieve organic materials based systems with better/higher photostability are demanded, because currently known and commercially available solutions could be still more efficient.

Another interesting issue regarding the control of optical properties of photonic materials, i.e. by external electric field, is the utilization of liquid crystals (LCs) as matrices for organic dyes. The latter ones functionalize whole system in order to achieve desired features. On the other hand, the long range order of LC molecules, as well as their high optical anisotropy, combined with their unusual fluid-like behavior, give the possibility to control the optical properties of such form of matrix. These include: matrix birefringence, ordering the dye transition dipole moments with respect to external excitation polarization or light-matter interaction leading to the nonlinear optical effects^10^. In the last years, researchers have provided strong support for the assertion that LCs have very unique optical and electro-optical properties^11^. It is worth to highlight features like: easily controlled birefringence, anisotropy, good/high photo-stability, and easy tuning of emitted light, especially by electrical field^12–14^. Due to abovementioned fluid-like behavior of LCs, there is a possibility to change their molecular arrangement in a very simple way. For example, by utilization of external electric or magnetic fields as well as stress or interact with spatially functionalized interfaces^15^. Birefringence can provide the significant changes of the optical properties for the thin volumes of liquid crystalline matter. One example of LCs utilization is the tunable dye lasers construction. According to the literature, LCs can serve as a matrix for many host-guest systems. Therefore, efficient stimulated emission phenomenon can be observed. This effect can be obtained among others, e.g. as a band-edge lasing in chiral LCs, or defect-mode lasers, depending of the type of chosen liquid crystal^16–18^. Dye doped LC systems in comparison with commonly used polymer matrices characterize similar or even lower pumping energy thresholds^19,20^. Moreover, some examples of the obtained stimulated emission phenomena with low energy threshold observed for LC and polymer combined matrices, were reported in the literature before^21^. In the case of polymer-liquid crystal blend it was possible to obtain lower energy threshold in comparison with homogenous matrix. Moreover, it was proven in literature that by playing with external factors, like magnetic or electric field, induced laser line wavelength in LC-based systems, can be tuned effectively. Therefore, fluorescence and STE linewidth with its threshold value as well as emission tunability controlled by external DC electric field in the investigated liquid crystalline system were investigated.

Several examples of laser devices utilizing LCs were shown in the literature^22^, especially in the context of electrically controlled laser emission^23^. LC lasers can have great potential in display technologies^24^, however, the number of organic dyes that can be successfully incorporated into liquid crystalline matrix, is limited. Therefore, there is a necessity to design and synthesize new dyes, which can be suitable for LCs matrices. Perylene derivatives dyes meet this condition. They have been widely reported in literature and became very important because of their excellent luminescent properties^25^, possible application in photovoltaics^26–28^ and other organic semiconducting devices^29^. It is thank to their high electron affinity and mobility^30^. Furthermore, perylene-based compounds are commonly used as the pigments^31^, and form aggregates in concentrated solutions. Some of them are able to create a monomolecular layer at the air/water interface using Langmuir-Blodgett technique. In such a way it is possible to control the dye orientation and molecular organization. Depending on the dye molecular structure J-, I- and H-aggregates can be formed in Langmuir and Langmuir-Blodgett films^32–35^. Especially J-aggregates are very attractive due to high fluorescence quantum yield and spectral range^36,37^. It is worth to mention that aggregates formation for typical laser dyes, mostly leads to concentration quenching effect^38^. Such aggregation is observed in LC matrices usually due to the low miscibility of many dyes in this kind of matrix. Therefore, only small number of laser dyes were successfully utilized for LC doping, i.e. coumarins^39^, pyrromethenes^40^ or DCM dye^41^. Moreover, formation of different types of aggregates or small crystals might be beneficial if consider photo-stability improvement. We can expect that increased rigidity can enhance the heat dissipation within the dye crystals, decreasing the impact of dye thermal decomposition on photo-degradation processes. The miscibility of molecular oxygen inside crystals is further less than in LCs, therefore it could be expected that quenching effect related to photo-oxidation phenomena also should be limited. It is worth to mention that system prevention from molecular oxygen also might be realized at the stage of LC cell preparation. In that way, the encapsulation strategy also might be applied. Moreover, the improvement of photo-stability can be achieved by the aid of multiple scattering effect, generated by dye crystals and LC inhomogenities, leading to photon transport realizing many different trajectories that do not exploit material in such a way that is for ballistic transport, when photons are propagating along particular, defined paths.

In this work we focused our attention on the optical properties of perylene derivative, namely the 3,4,9,10-tetra-(7-alkoxy-carbonyl) perylene (TACP). In our studies TACP compound plays a role of the active matter (guest) in a nematic liquid crystalline matrix (host) characterizing negative electrical anisotropy. Macroscopically oriented liquid crystalline domains cause particular orientation of the small-guest molecules. In our study we investigated another approach - doping the LC matrix with novel dye, characterized by high emission quantum yield in aggregated form which does not require high miscibility of the used dye and might be beneficial for construction of LC based laser devices. The light amplification investigation has been conducted and following parameters were examined, i.e. influence of the applied electrical field or different excitation energy density at the spectral properties, especially for the dye photo-degradation process.

### Materials and sample preparation

**Figure 1:**
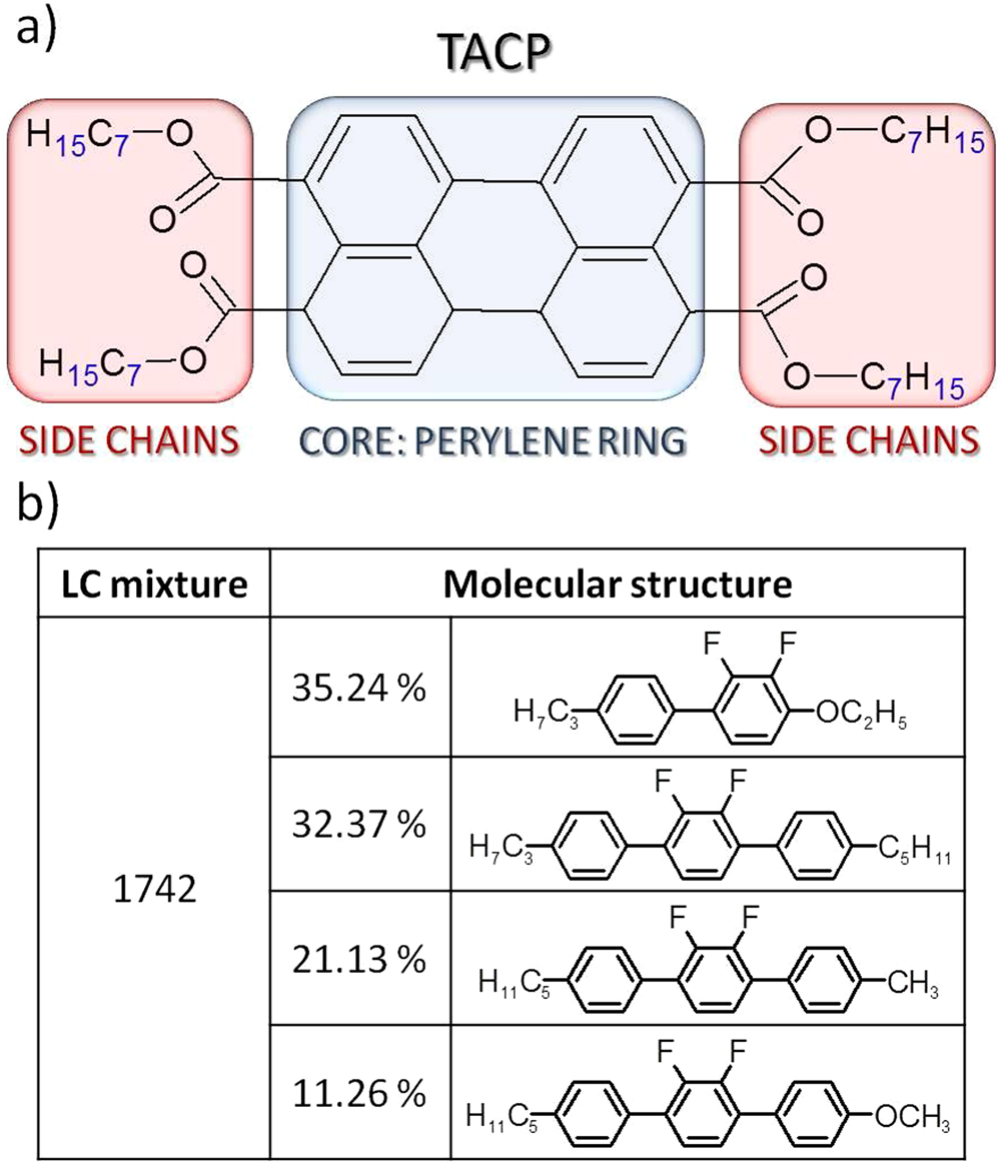
Molecular structure of 3,4,9,−10-tetra-(7-alcoxy-carbonyl)-perylene (acronym: TACP); structure’s core - perylene ring and four aliphatic side chains marked on blue and red color, respectively (**a**) molecular composition of the 1742 liquid crystalline mixture (**b**).

The studied luminescent dye (3,4,9,-10-tetra-(7-alcoxy-carbonyl)-perylene – (TACP)) structure presented in Fig. 1a is based on the perylene ring (characterized by naphthalene units) with attached four alkyl chains consist with seven carbon atoms. In normal conditions, after synthesis it occurs as the yellow solid state powder. The compound shows high fluorescence quantum yield in diluted solutions and is practically independent of alkyl chain length (QY = 87.5)^42^ and for polycrystalline powder (QY = 18.2)^43^. The green fluorescence lays in maximum sensitivity of human eye. The dye was synthetized and purified by thin layer chromatography (TLC) at Lodz University of Technology. The detailed synthesis route is presented elsewhere^44,45^.

The TACP dye was incorporated into nematic liquid crystalline mixture **1742** characterizing the negative dielectric anisotropy (∆ε = −3.250) due to the occurrence of fluorine atoms, laterally substituted to rod-like molecules composing the nematic mixture^46^. **1742** was purchased (obtained) from Prof. Dabrowski (MUT, Warsaw, Poland). The composition of **1742** mixture is presented in Fig. 1b. The phase transition temperatures between solid/nematic and nematic/isotropic phases for this mixture are defined at: T_nem_ = 25 °C and T_iso_ = 87 °C, respectively. More details about the mixture parameters can be found in ref.^47^.

The liquid crystal cell is hand-made and consists of two 1 mm thick glass plates (20 × 30 mm^2^) with Indium Tin Oxide (ITO) transparent electrodes. The ITO electrodes were covered by 1,2,3-trimethyl trisilane from (4% chloroform solution – 24 hours incubation) in order to create the homeotropic orientation of **1742** liquid crystal layer. Two glass plates oriented antiparallel were separated by a 60 µm thick strips of Teflon and fixed by epoxy glue on two shorter sides. The liquid crystal cell was filled with **1742** mixture by capillary forces. In such way, a mono-domain liquid crystalline sample with homeotropic orientation of LC was created. The molar concentration of TACP in the **1742** LC matrix (1.5 × 10^−2^ mol/dm^3^) was high enough to cause the dye re-crystallization process.

### Experiment

**Figure 2:**
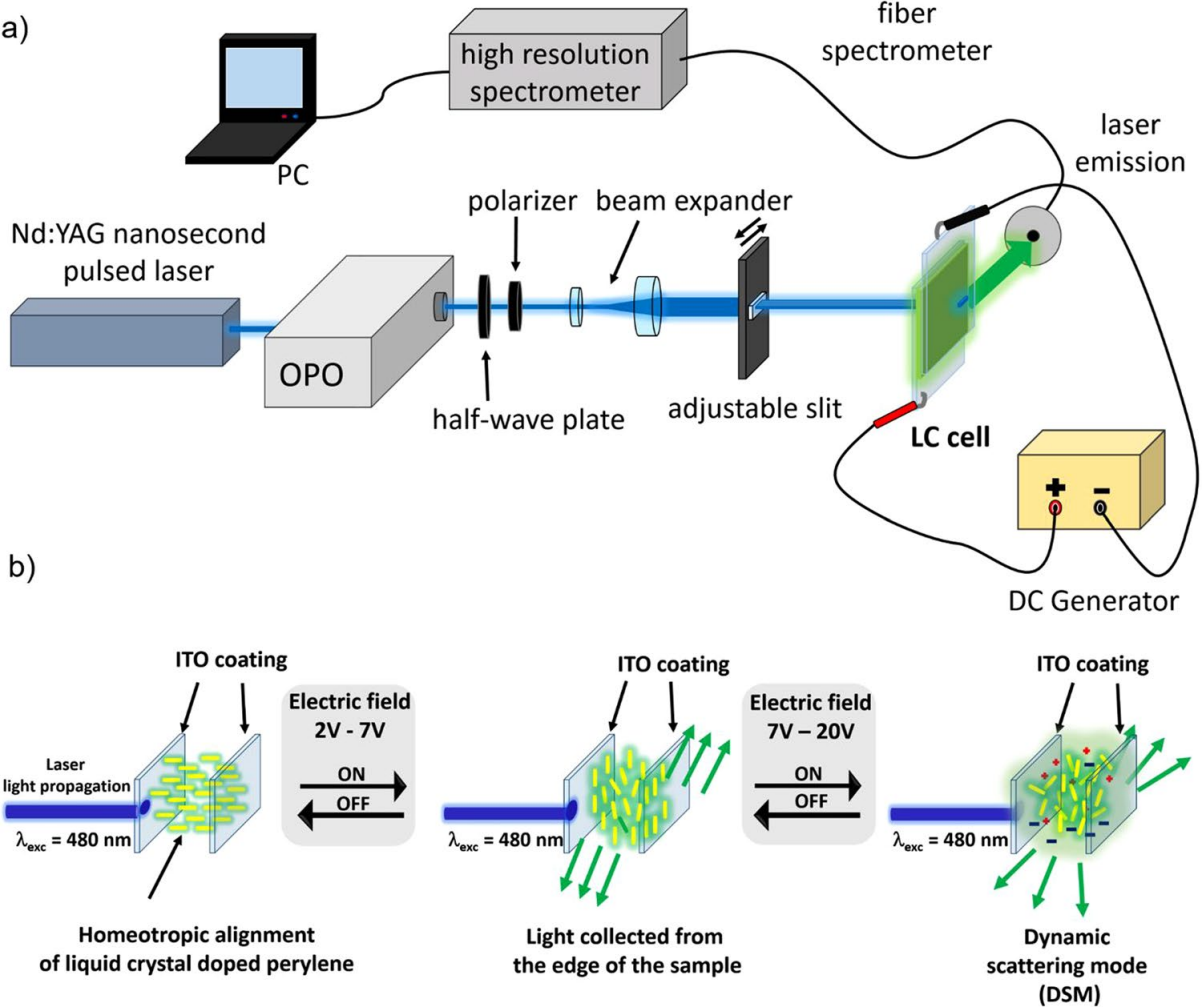
Experimental set-up for STE measurements. The emitted light is collected by optical fiber from the edge of the sample excited by stripe-shape laser beam. (**b**) The scheme representing transition of the liquid crystal molecules alignment and light propagation direction induced by applied external electric field.

For measurements of Stimulated Emission (STE) we have assembled the following experimental set-up which is schematically shown in Fig. 2. The vertical linearly polarized light of the wavelength λ = 480 nm coming from optical parametric oscillator (Horizon, high efficiency mid-band OPO by Continuum) pumped by the tripled in frequency Nd:YAG laser fundamental line (Surelite II, pulse duration 6 ns, 10 Hz repetition rate) was incident normally at the sample. Light beam passed through the polarizer, diaphragm, half-wave plate and system of lenses, adjustable slit with the converging cylindrical lens, forming an extended stripe of the light with adjustable length. The dimension of the stripe was chosen to 4 mm × 0.5 mm (length × height). Pump light energy was measured with a calibrated laser energy meter (Coherent Field Max II coupled with J-10MB-HE sensor). STE emerging from the edge of the sample was collected by an optical fiber and analyzed with high resolution (0.1 nm) spectrometer (Shamrock 163) coupled with a computer. Additionally, the DC power supply (NDN DF1750SL3A) connected to the ITO electrodes on the glass plates of the LC cell was used in order to generate an electric field across the sample.

Prior to the stimulated emission measurements, the emissive and absorptive properties of TACP dye in LC matrix were studied. The photoluminescence measurements were carried out using the Hitachi F-4500 fluorescence spectrophotometer within the range of 350–650 nm at room temperature. The spectral resolution was set to 1 nm for both excitation and emission spectra. For the UV-VIS spectroscopic measurements we have used LC cells without applied voltage to the sample (homeotropic alignment).

## Results and Discussion

**Figure 3:**
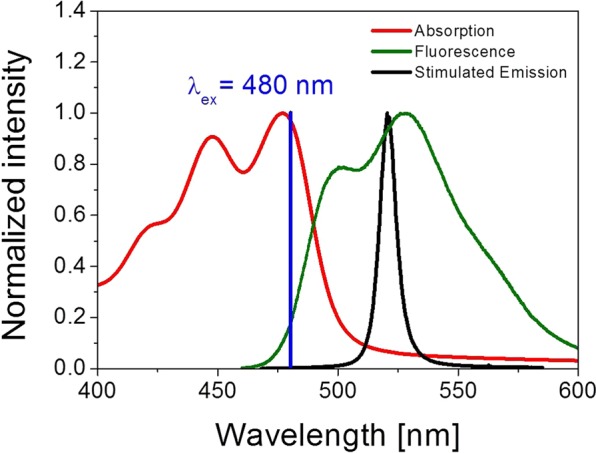
Absorption (red line), fluorescence (green line) and Stimulated Emission (black line) spectra measured for TACP/1742 mixture in LC Cell. Additionally, incident laser line (blue color) is showing excitation line λ_ex_ = 480 nm, the intensity of pumping beam is equal to I_pump_ = 9.7 mJ/cm^2^.

The normalized excitation and fluorescence spectra of **1742** mixture with TACP dye in LC cell is shown in Fig. 3. The full width at half maximum (FWHM) of fluorescence emission (green line) is about 69 nm and the maximum is centered at around 528 nm. The emission is showing multi-band structure with local maximum at around 500 nm (blue shifted with respect to global maximum) and a small hump at red-shifted shoulder at around 565 nm. Similarly, the excitation spectrum (red line) is also showing multiband shape with maxima located at 420, 448 and 477 nm, for which the last one is also the most intense one. Such wide excitation range gives the possibility to excite sample with light from blue and blue-green region of visible light. In Fig. 3 the excitation line from OPO was also shown as blue line.

**Figure 4:**
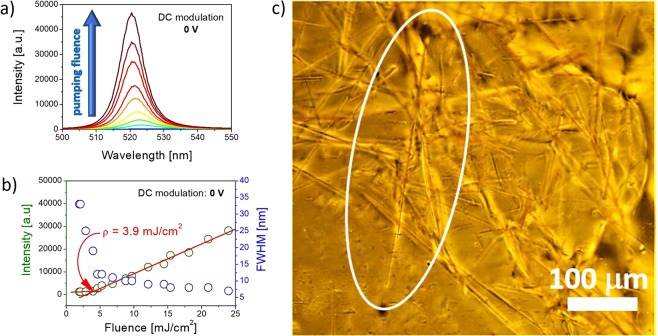
(**a**) The stimulated emission spectra obtained for the **1742** mixture doped with TACP dye in the LC cell; (**b**) The STE threshold and FWHM characteristic in function of pumping energy density. The measurements were obtained for the stripe shape of laser beam with length = 4 mm, height = 0.5 mm and excitation wavelength λ = 480 nm. (**c**) The microscopic image presenting the dye aggregation forming microcrystals.

The results of stimulated emission measurements in function of excitation energy density and without applied voltage to the sample, are presented in Fig. 4a. One can clearly see a characteristic narrowing of the fluorescence line width above the STE threshold. For the reported LC-perylene system we observe decrease of the luminescence bandwidth from Δλ_FWHM_ ≈ 69 nm for the lowest chosen excitation energy density (below gain threshold) to Δλ_FWHM_ ≈ 7 nm for STE light emitted from the sample and collected by the fiber spectrometer, what is shown in Fig. 4b. The estimated excitation intensity threshold of STE is about 3.9 mJ/cm^2^. What is very interesting, while intensity of emission increases, the maximum of the emission band is blue-shifted. Such phenomenon might be explained in the terms of solid state structures formation and excitonic nature of emission. While excitons diffusion depends on material parameters and is invariant on excitation intensity, excitons cannot dissipate as much energy when pumped with high intensity light as for low pumping rates, because the population inversion state is being established much faster, what results in faster recombination and blue shift of emission. Indeed, the TACP dye crystals appears in the sample as the long and thin needles, what was evidenced by optical microscopy investigation. The micrograph showing TACP crystals is presented in Fig. 4c. The other explanation of blue-shift might be linked to increasing photo-bleaching effect caused by higher pumping intensity, which in turns decrease the reabsorption of emitted light.

**Figure 5:**
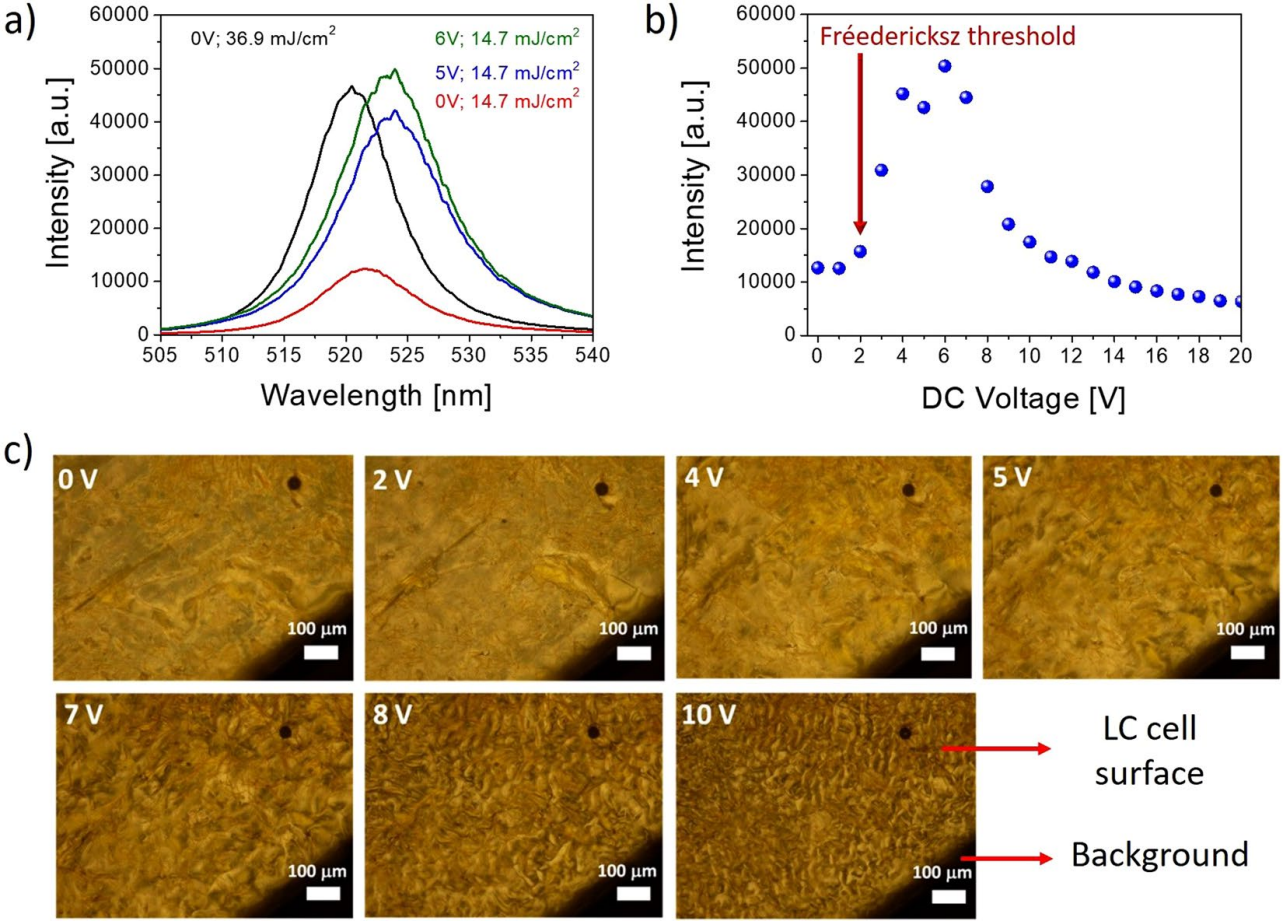
(**a**) The dependence of the STE peak intensity and applied to the cell DC voltage. The pumping fluence was set at 14.7 mJ/cm^2^. (**b**) STE bands upon the same (colorful lines) and much higher (black color) excitation intensity with visible blue- and red-shifted positions. (**c**) A set of microscopic images acquired in the crossed polarizer mode (optical microscope) showing the changes of liquid crystal texture when the DC voltage is applied. In the presented photos, edge of sample is visible as a dark field. The scale bar is: 100 μm.

Influence of DC electric field applied to the sample on light enhancement is presented in Fig. 5a. The measurements were conducted in the range of 0–20 DC voltage and for the pumping fluence above the STE threshold, set to 14.7 mJ/cm^2^. We can observe the electric field dependence resulting in significant increase of light emission above the Freedericksz threshold (about 2V). For the value of 6 volts we have obtained six times higher light emission than for the conditions without applied voltage. For the higher voltage values the emission intensity decreases. Such behavior is strictly related to the LC mixture properties. The **1742** LC mixture is composed of nematic components characterized in general by the negative dielectric anisotropy (∆ε = −3.250). The molecules of the nematic LC phase with negative dielectric anisotropy (measured at low frequencies) characterize the dipole moments oriented perpendicular to their long axis. According to this fact, molecules above the Freedericksz threshold tend to align their long molecular axes perpendicularly to the direction of electric field applied to the sample. Parallel-like position of dipole moment for the range of 6–8 volts and polarization state of laser light used for the sample excitation, give the optimum conditions for light propagation and dye excitation, observed as the enhancement of STE intensity, what was schematically shown in Fig. 2b. When the voltage is further increased, the strong turbid movements of the LC molecules are induced by the ionic current flow leading to the dynamic light scattering (DLS) effect. Such electro-hydrodynamic instability disturbs effective light wave-guiding process, what is observed as the light intensity decrease collected from the sample edge. The DLS phenomenon was evidenced by polarized optical microscopic measurements. The exemplary images showing LC texture change upon DLS process, obtained by cross-polarizer microscope, are shown in Fig. 5c. This process becomes significantly visible after reaching 7V of the applied voltage, what is in accordance to the previous STE intensity investigations.

The comparison of chosen emission spectra obtained in the process of light amplification, with and without applied external electric field to the sample are presented in Fig. 5b. For the presented examples of spectra, when no voltage is applied, the intensity of emission rises nearly fivefold when pumping fluence becomes changed from 14.7 mJ/cm^2^ up to 36.9 mJ/cm^2^. Similar increase of STE intensity for pumping beam fluence, kept at the level of 14.7 mJ/cm^2^, can be achieved by applying the voltage of 6 volts to the sample. However, in such a case slight red-shift of emission spectra can be observed. Increased STE intensity with applied voltage may be also beneficial from the photo-stability point of view. The emission signal can be kept at the same level of intensity even if pumping fluence is decreased by 2.5 times. In such conditions the material is likely to be less exploited than in case of higher excitation intensities. Moreover, it is worth to notice that such control of emission intensity might be realized by applying relatively low voltages – just in the range of 4 to 8V.

**Figure 6:**
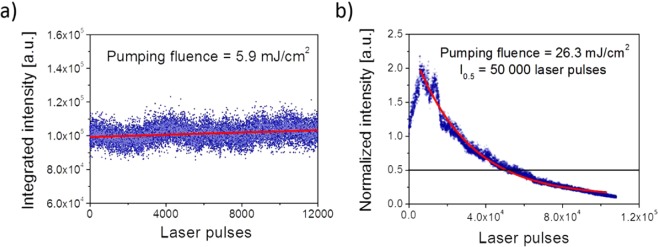
Photostability investigation obtained for the pump fluence (**a**) I_ex_ = 5.9 mJ/cm^2^ and (**b**) 26.3 mJ/cm^2^.

Additional, the advantage coming from utilization of emissive crystals together with LC mixture is the high photo-stability of resulting material. We manage to conduct two experiments regarding to investigate the material’s photo-stability. At first, we set the energy density of exciting light at the level of 5.9 mJ/cm^2^ (slightly above the STE threshold) and repetition frequency equal to 10 Hz. After 12 000 laser pulses we have not observed any STE signal decrease, what evidenced high temporal stability of obtained material. Secondly, we changed the illumination place on the sample and we irradiate it with much higher energy density, at this time equal to 26.3 mJ/cm^2^. The STE intensity has decreased by half after 50 000 laser pulses. The results of temporal stability measurements are shown in Fig. 6a,b for both excitation energy densities, respectively.

What is interesting, in the second case it can be clearly seen that surprisingly at begging the intensity of STE is rising, reaching its maximal value after around 8000 laser pulses, which is almost twice higher than its initial value. Such behavior may be explained, for example, in the terms of the liquid crystalline fluid-like behavior. In case of the high intensities of pumping light generated by nanosecond pulse laser, the horizontally oriented electric field can interact with TACP transition dipole moments and thus causes molecular orientation along the electric field vector of incoming light. Consequently, after each laser pulse, pumping light becomes absorbed by increasing number of dye molecules resulting in more efficient pumping. This process is opposite to the expected typical photo-degradation trend, therefore in Fig. 6b it can be observed the sharp peak of intensity owing to the occurrence of two separated exponential processes, leading to increase and decrease of light intensity, respectively. Therefore, the boost of light intensity as a result of increasing transparency generated by dye photo-degradation process (dye diffusion, oxidation, thermal decomposition etc.) can be ruled out since the emission intensity vs. initial laser pulse number dependency should have smooth *plateau* before reduction of intensity value, opposite to the sharp peak visible in Fig. 6b. The second explanation might be based on the changes of scattering conditions generated by intense laser light. In case of scattering feedback whatsoever changes of scattering conditions can strongly affects lasing performance. Such changes may occur as a result of local heating disturbing the order parameter of liquid crystal and/or as the result of heat driven mass transport. Additionally, TACP crystals also provide light scattering and any optical damage, changing their size and structure, must lead to the changes of scattering conditions. According to this mechanism the increase of light intensity might be seen as a consequence of establishing more favorable light scattering conditions, supporting random feedback, while decrease of signals is strictly linked to the photo-degradation processes. Both scenarios are our speculations and requires further, more sophisticated and strictly oriented towards those phenomena studies, which are out of scope of the present article.

## Conclusions

We have shown that introduced in this manuscript strategy of incorporating dyes like TACP, exhibiting the strong emission in aggregated form, into LC matrices might be beneficial for the emissive LC materials. In general we can conclude that dyes which are not strongly affected by concentration related quenching (i), showing aggregated induced emission (ii) or aggregation induced enhanced emission (iii), might be superior among the other dyes according to obtainment of photo-stable luminescent organic materials. We have shown that in our particular case the re-crystallization process in small scale does not affect the liquid crystal phase, therefore all benefits coming from utilization of LC are sustained. It means that such parameters like emission intensity as well as STE energy thresholds might be controlled by external electric field. Such feature is desired for construction of opto-electronic devices. Moreover, according to previous conclusion, the appearance of highly emissive crystals in LC phase increases the photo-stability of laser system leading to the even almost lack of photo-degradation processes, when the system is operating around the threshold level. What is most advantageous, the re-crystallization strategy is still capable to be used together with other methods of photo-stability improvement like, i.e. encapsulation, or cell preparation in oxygen free atmosphere. Merging all of the mentioned approaches the resulting laser device can exhibit sufficient photo-stability for commercial requirements.
